# Large-scale human skin lipidomics by quantitative, high-throughput shotgun mass spectrometry

**DOI:** 10.1038/srep43761

**Published:** 2017-03-07

**Authors:** Tomasz Sadowski, Christian Klose, Mathias J. Gerl, Anna Wójcik-Maciejewicz, Ronny Herzog, Kai Simons, Adam Reich, Michal A. Surma

**Affiliations:** 1 Medical Faculty, Dresden University of Technology, Dresden, Germany; 2 Lipotype GmbH, Dresden, Germany; 3 Department of Dermatology, Venerology and Allergology, University of Medicine, Wroclaw, Poland; 4 Max Planck Institute for Molecular Cell Biology and Genetics, Dresden, Germany

## Abstract

The lipid composition of human skin is essential for its function; however the simultaneous quantification of a wide range of stratum corneum (SC) and sebaceous lipids is not trivial. We developed and validated a quantitative high-throughput shotgun mass spectrometry-based platform for lipid analysis of tape-stripped SC skin samples. It features coverage of 16 lipid classes; total quantification to the level of individual lipid molecules; high reproducibility and high-throughput capabilities. With this method we conducted a large lipidomic survey of 268 human SC samples, where we investigated the relationship between sampling depth and lipid composition, lipidome variability in samples from 14 different sampling sites on the human body and finally, we assessed the impact of age and sex on lipidome variability in 104 healthy subjects. We found sebaceous lipids to constitute an abundant component of the SC lipidome as they diffuse into the topmost SC layers forming a gradient. Lipidomic variability with respect to sampling depth, site and subject is considerable, and mainly accredited to sebaceous lipids, while stratum corneum lipids vary less. This stresses the importance of sampling design and the role of sebaceous lipids in skin studies.

The primary function of the largest organ of the human body - skin - is to keep in what is inside and keep out what is outside. This functionality, its physiology and pathophysiology are derivative of structure and composition of its topmost layer, the stratum corneum. The SC consists of a layered meshwork of corneocytes sealed with lipids arranged in lamellar fashion. The most abundant constituents of the lipid matrix are ceramides (Cer), cholesterol (Chol) and free fatty acids^1^. Other classes found in SC include triacylglycerol (TAG), diacylglycerol (DAG) and cholesterol esters (CE)^2^. Lipidomic studies of skin have demonstrated that age, gender, ethnicity, and season of the year affect the skin lipid composition^3^. Likewise, alterations of lipid profiles were linked to dermatological and systemic diseases, like atopic dermatitis^4^,^5^, hereditary ichthyosis^6^ or Netherton syndrome^7^.

Early pioneering thin layer chromatography studies discerned 8 ceramide sub-classes^8^. The advent of mass spectrometry coupled with chromatographic separation of analytes (i.e. LC-MS) offered much increased sensitivity. This allowed further differentiation of ceramides into 12 ceramide sub-classes and identification of other lipid classes^9^,^10^,^11^,^12^. These 12 ceramide sub-classes are defined by a combination of four different types of sphingoid bases (dehydrosphingosine, sphingosine, phytosphingosine and 6-hydroxy sphingosine), referred to as long chain base (LCB) with three different types of acyl chains (non-hydroxy, alpha-hydroxy and esterified omega-hydroxy fatty acid (FA))^1^.

A more recent approach to skin lipidomic analysis utilizes shotgun mass spectrometry, where the sample extract is subjected to mass spectra acquisition without prior chromatographic separation (direct infusion)^13^,^14^. This technology was applied for the analysis of non-hydroxy and alpha-hydroxy ceramides, but it did not allow for absolute quantification or for the distinction between all ceramide sub-classes.

In comparison to shotgun, mass spectrometry involving chromatographic separation of analytes is characterized by increased sensitivity due to the lowered spectra complexity, which together with additional information about retention times makes them suitable for the discovery of new, even low abundant lipids^10^. The lack of chromatographic separation prior to analysis in shotgun mass spectrometry requires high-resolution instruments, as spectra are very complex, which also necessities refined data processing^15^. On the other hand, it makes this technique more suitable to cover a higher number of lipid classes, which would otherwise necessitate different separation conditions and longer analysis time in chromatographic approaches.

However, the central advantage of shotgun mass spectrometry resides in its high-throughput capabilities. We previously reported the development of a shotgun lipidomics platform for the analysis of blood plasma permitting the comprehensive quantification of lipids in hundreds of samples^16^. The capacity to analyze a high number of samples allows screening, biomarker, intervention, and mode-of-action studies, facilitating a much-needed increase in the statistical power of lipidomic results. High throughput is also a prerequisite for any prospective clinical setting of skin lipidomics as well as for derma-pharmacological and cosmetic applications.

In order to exploit fully the high-throughput potential of shotgun lipidomics for skin samples, the sampling procedure itself has to fulfil certain requirements. Firstly, it needs to deliver reproducibly a sufficient amount of material to allow comprehensive lipid coverage. Secondly, the variation of lipid composition with respect to sampling site as well as sampling depth should be controlled, especially if insight into physiological processes is sought. Furthermore, the sampling procedure should neither introduce exogenous substances into the sample, nor remove (or suppress) analytes.

Several skin-sampling techniques are employed so far, most fulfilling only some of the above criteria at a time. Scrape biopsy for instance delivers abundant amounts of sample with minimal contaminants, but neither the exact sample amount, nor sampling depth can be easily controlled. *In situ* extraction (the so-called “cup method”^17^) offers clean and direct extraction, but does not have high-throughput capabilities and is potentially taxing for the subject. Tape-stripping offers control of sampling position, depth and sample amount. However, the extraction of polymeric adhesive tape with organic solvents can potentially introduce background.

In this paper we present a shotgun mass spectrometry-based lipidomic method compatible with the most convenient sampling method – tape stripping – and characterized by broad lipid coverage, including unambiguous identification of lipid species belonging to all 12 ceramide classes, as well as di- and triacylglycerols, cholesterol and its esters. All lipids are absolutely quantified by inclusion of internal standards. In combination with automated lipid extraction and acquisition, this method achieves unprecedented high-throughput.

With this method, within days we performed to our knowledge the largest (104 subjects; more than 268 individual samples analyzed in total) stratum corneum lipidomic study to date. We investigated (1) the dependence between SC lipid profiles and sampling depth; (2) determined intra-individual variability of the SC lipidome by analyzing 14 different sampling sites; and (3) assessed inter-individual variability by analyzing SC lipidomes of 39 males and 65 females of different age.

## Materials and Methods

### Nomenclature

Lipid species are annotated according to their molecular composition as follows: [lipid class]-[sum of carbon atoms in LCB and FAs]:[sum of double bonds in LCB and FAs];[sum of hydroxyl groups in LCB and FA] (e.g., EOS 70:3;2 denotes omega-hydroxy-shpingosine with a total length of its LCB and FA of 70; with 3 double bonds and 2 hydroxylations in total). For lipid sub-species, the individual acyl chain composition according to the same rule is given (e.g. 18:1;0–24:2;0), with the first entity denoting a sphingoid base (LCB) and the second a fatty acid (FA), in case of ceramides. Ceramides naming convention was adopted from^18^.

### Materials

Methanol, propan-2-ol, chloroform, acetyl chloride and ammonium acetate were of analytical grade. Deuterated NS D3 (36:1;2) (cat# 2201) and EOS D9 (68:3;2) (cat# CUS9530) were purchased from Matreya LLC. Deuterated TAG D5 (cat# 110544) and DAG D5 (cat# 110538), and CE(20:0;0) (cat# 110870) were purchased from Avanti Polar Lipids.

### Skin sampling

All sampling from human subjects occurred with informed consent in accordance with the Declaration of Helsinki and was approved by the Bioethical Committee of the Wroclaw Medical University (decision number 406/2015).

Scrape biopsy samples were collected from the calves of one subject by careful scraping of the topmost skin layers with a scalpel directly into a tube.

The sampling site for tape-stripping was prepared by removing the topmost SC layer with a large CUDERM D-Squame sampling disc (D100). A discs was pressed on the sampling site for 15 s with a D-Squame pressure instrument (D500) providing uniform pressure (225 g/cm^2^), and removed with one fluent motion. Next, in order to collect the actual sample, a small D-Squame stripping disc (D101) was pressed with the pressure instrument to the prepared site again for 15 s, removed and placed into a tube. Samples were stored at −20 °C until extraction.

The sample amount collected with stripping discs was determined gravimetrically using a Radwag Microbalance Type MYA 5.3Y. To this end, 42 volar forearm samples of 6 subjects were collected according to above protocol, while stripping discs were weighed directly before and after sampling, the difference being equal to the weight of skin collected.

For the investigation of lipidome variability with respect to sampling depth, triplicate samples from adjacent positions on left volar forearm from one female and one male subject were collected layer by layer – including the topmost – until the 20^th^. After the 10^th^ layer every second layer was sampled, and the remaining layers were discarded. Thus, 90 samples were collected in total. All these sequentially tape-stripped samples belonged to the stratum corneum proper consisting of corneocytes embedded in a lipid matrix^19^.

For the assessment of intra-individual skin lipidome variation triplicate samples from adjacent positions on skin were collected from each sampling site. Samples were collected from one male and one female at 14 different sites (forehead, cheek, pectoral region, abdomen, groin, thigh, heel sole, central calf, shoulder blade, buttock, palm, outside of hand, top side of foot and volar forearm). Laterally symmetric sites were all sampled from the left side of the body.

For the inter-individual lipidome variation 65 females and 39 males of different age (20–89) were sampled from the volar forearm, totalling 104 samples.

Each analytical batch of samples was complemented by blank samples each containing a sampling disc without skin.

### Lipid extraction

Lipid extraction of tape-stripping samples containing one stripping disc each and scrape biopsy samples alike was carried out in 2 mL polypropylene tubes where 900 μL of methanol including internal standards was added to each sample. The samples were shaken at 1400 rpm at 4 °C for one hour. Thereafter, extracts were transferred to a multi-well plate and dried in a speed vacuum concentrator. Dried extracts were re-suspended in an acquisition mixture of 7.5 mM ammonium acetate in chloroform:methanol:propan-2-ol (1:2:4, V:V:V). For cholesterol, the dried extract was acetylated^20^, then dried again and re-suspended in the above acquisition mixture. All liquid handling steps were performed using Hamilton Robotics STARlet robotic platform with Anti Droplet Control for pipetting of organic solvents.

### MS data acquisition

Samples were analyzed by direct infusion with a QExactive mass spectrometer (Thermo Scientific) equipped with a TriVersa NanoMate ion source (Advion Biosciences) in a single acquisition for both positive and negative ion modes with a resolving power of R_m/z = 200_ = 280000 for MS and R_m/z = 200_ = 17500 for MSMS. MSMS fragmentation was performed at normalized collision energy of 35% and was triggered by an inclusion list encompassing corresponding MS mass ranges^16^. Both MS and MSMS data were combined to monitor CE, DAG and TAG ions as ammonium adducts and all ceramide sub-classes as acetate adducts. Precursor ions and confirmatory MSMS fragments as reported previously^10^,^13^,^14^ are summarized in Supplementary Table 1. Cholesterol was identified in a separate acquisition as cholesterol-acetate after a derivatization procedure^20^.

### Lipid identification and quantification

The resolving power used allows for identification of lipids based on precursor masses^21^. Combining MSMS fragmentation data with the high resolution MS precursor data followed by isotopic correction (type I and II according to the strategy described previously^16^,^22^), permitted structural elucidation of lipid molecular species. It increased the identification specificity, but also made it possible to unambiguously distinguish all 12 ceramide sub-classes; including NP/AdS and NH/AS pairs.

Internal standards used in this study were chosen not to be natively present in skin samples. Per sample 42 pmol of EOS D9 68:3;2 (18:1;2, 32:0;0, 18:2;0), 14 pmol NS D3 36:1;2 (18:1;2, 18:0;0), 50 pmol DAG D5 34:0;0 (17:0;0, 17:0;0), 100 pmol CE 20:0;0, 1000 pmol cholesterol D6 and 100 pmol TAG D5 51:0;0 (17:0;0, 17:1;0, 17:0;0) were delivered as internal standards. Quantification was conducted via normalization of the isotopically corrected intensity of the monoisotopic peak of each native species to the isotopically corrected intensity of the monoisotopic peak of the internal standard. The quantities of lipid molecular species were calculated from ratios between their respective characteristic MSMS fragments, as described previously^16^. Non-hydroxy and alpha-hydroxy ceramide sub-classes were normalized to deuterated NS, whereas omega-hydroxy ceramides were normalized to deuterated EOS. Quantitative lipidomic data of the study can be found in Supplementary Table 2.

### Data analysis and post-processing

Data were analyzed with in-house developed lipid identification software based on LipidXplorer^23^,^24^. Only lipid identifications with measured mass deviations below 3 ppm from the theoretical mass for MS and 8 ppm for MSMS peaks in scans where lock mass was available, and 5 and 12, respectively, where it was not; signal-to-noise ratio greater than 5; and an amount at least 5-fold higher than in corresponding blank samples were considered as positive hits, yielding 862 unique lipids across all samples from all experiments. Further, unless stated otherwise, only lipids present in at least two replicates and above 2 pmol per sample were included in subsequent data analysis, resulting in 509 unique lipids. Data visualization, linear regression (linear least squares method), and correlation (two-tailed Pearson and Spearman correlation) calculations were performed on Prism6.0 h software (GraphPad Software, Inc.). Where it does not lower figure clarity, individual data points are shown^25^.

Statistical models were trained with the R Environment for Statistical Computing (R version 3.3.2 (2016-10-31)), by the caret package (version 6.0–73)^26^, which in turn uses the randomForest package (version 4.6–12)^27^. For statistical modeling only lipids present in at least 50% of samples per cohort were used. If a lipid was present in one cohort, it was also included for other cohorts, even if its occurrence rate was lower. During model training with 5 times repeated 10 times cross validation, the resampled data were preprocessed by centering and scaling, missing values were imputed with the median, and near zero variance predictors were removed.

## Results

### High throughput-compatible skin sampling and sample processing

In order to take advantage of the quick spectra acquisition capabilities of shotgun mass spectrometry, the sampling procedure and all other sample-handling steps were optimized for speed, throughput and convenience utilizing automation whenever possible. Automation of procedures also benefits in higher reproducibility^28^.

**Figure 1:**
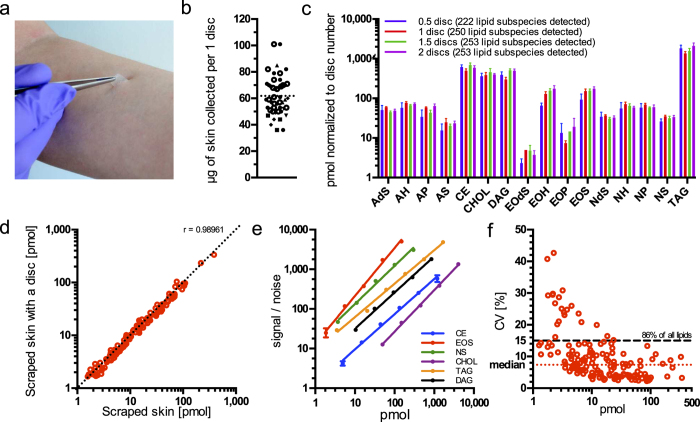
Method validation. (**a**) Tape-stripping skin collection method. A disc with adhesive is attached to skin, pressed and peeled off. (**b**) Amounts of skin collected with 1 stripping disc. Different shapes represent 6 donors, total n = 48; dotted line denotes the mean. (**c**) Comparison of lipid class amounts normalized to number of stripping discs extracted. Data show the mean of 5 independent experiments and error bars the standard deviation. Only lipids present in all replicates were considered. (**d**) Pearson Correlation of averaged lipid subspecies amounts determined for skin samples with and without stripping disc present. Every point represents the averaged amount of the individual lipid subspecies quantified from 5 independent extractions and acquisitions; only lipids present in all replicates were considered. Correlation coefficient (r) is given. The dashed diagonal represents slope of 1. (**e**) Dynamic ranges determination per lipid class. Internal standards were spiked in the skin and the signal to noise recorded. A linear regression fit is shown. Data points show the mean of 5 independent experiments and error bars the standard deviation. (**f**) Correlation between coefficients of variation and mean lipid amounts from 10 independent acquisitions and quantifications of pooled extracts from 3 different skin samples. Each point represents the individual lipid subspecies; only lipids present in all replicates were considered.

Tape-stripping was used as it was simultaneously the most convenient and non-invasive sampling method (Fig. 1a). It also had the additional advantage of allowing for control over the sampling depth (by collecting the appropriate stratum corneum layer by sequential stripping) and collecting comparable sample amounts. As surface furrows might affect the amount of material collected^29^, we gravimetrically determined the reproducibility of sample amount collected via tape-stripping of the second layer with one stripping disc to be 62 ± 14 μg (mean ± s.d.) (Fig. 1b). Extraction of lipids with commonly used organic solvents from a polymeric tape with adhesive (i.e. stripping disc) was complicated because established methods utilizing chloroform^30^,^31^ or methyl *tert*-butyl ether^32^ interfered with tape constituents, physically dissolving them. We found, however, that methanol extraction successfully used for skin lipidomics in previous studies^11^ allowed for efficient extraction of skin lipids from stripping discs without compromising analysis quality, even without prior chromatographic separation of extracts.

Next we investigated whether the sample amount delivered by one stripping disc is suitable for lipid analysis. We found that the lipid amounts quantified from 0.5, 1, 1.5 and 2 discs stripped from the SC from one subject were linearly proportional to the number of discs used (Fig. 1c). Some of the ceramide sub-classes (EOdS, AS or EOH) were not proportionally represented when only half a disc was used. This was due to low abundant species falling below the limit of detection, as also reflected by a lower number of lipids detected in total (222 in comparison to 250–253 in other sampling amounts). However, the sample amount collected by one stripping disc ensured that all lipid classes were measured proportionally and maximum coverage of lipid species was achieved. Therefore, one stripping disc per sample was used for all other experiments.

In order to rule out a negative influence of stripping disc constituents on the analysis, we compared the results obtained from skin samples analyzed with and without a stripping disc present. To this end, we collected skin by scraping and divided it into individual samples of a weight similar to the amount collected with one stripping disc (77 ± 2.5 μg per sample, mean ± s.d.). To 5 samples one clean stripping disc was added and to the remaining 5 none. All ten were extracted and analyzed as described, and the lipid quantities were compared (Fig. 1d). Evidently, even in the presence of a stripping disc, lipid amounts were virtually identical to amounts in samples without disc (Pearson correlation coefficient = 0.99, P < 0.0001). This result shows that stripping discs do not influence lipid quantification.

### Method sensitivity and dynamic range

Due to the wide concentration range of analytes encountered in lipidomics analyses, it is essential that the analytical method spans a large dynamic range and offers sufficient sensitivity to allow for the quantitative analysis of molecules in the low and high concentration regime. To determine these parameters, we added increasing amounts of lipid class-specific reference lipids into tape-stripped skin samples, and we recorded how the acquired signal correlates with the amount (Fig. 1e). Lipid references used for this assessment needed to be distinguishable from endogenous lipids; therefore this analysis was limited to classes for which a proper reference lipid was available.

Linear dynamic range was defined as the concentration range at which the linearity of signal-to-noise values to pmol amount and the slope of the resulting function were close to 1 (slope from 0.85 to 1.21, depending on the lipid class (Supplementary Table 1)). This implies that changes in concentration of a given lipid will result in directly proportional changes in signal. The limit of quantification of most lipid classes lies in the low pmol range. Only for cholesterol it is about 50 pmol. However, this is sufficient since cholesterol is typically in the range of hundreds of pmol per skin sample. The linear dynamic range spans 1.8 (for NS, EOS, DAG and cholesterol) and 2.5 (for TAG and CE) orders of magnitude. Thus, the method is capable of measuring at least a hundred-fold change reliably.

### Reproducibility

Reproducibility of the method was assessed by analyzing lipids in samples collected from three different subjects and pooled after extraction. Pooled extracts were analyzed and quantified independently (n = 10), and the coefficient of variation (CV) of all quantified lipid sub-species was plotted against their respective amounts (Fig. 1f). Median CV was 7.37% with 86% of all lipid subspecies varying by less than 15%. As expected, there is an inverse correlation between CV and lipid amount^16^,^33^. Obtained reproducibility parameters place majority of quantified lipids well below the 20% CV threshold that is commonly used for *in vitro* diagnostic assays^34^. Together with the method’s sensitivity and dynamic ranges reported above, shows that skin lipids can be reliably and reproducibly measured in tape-stripped samples, meeting the criteria of a good analytical method^34^.

### Deep sampling by sequential tape-stripping

To check how skin lipidome changes with the depth, we collected samples from the left volar forearm from one female and one male through sequential layer by layer stripping – including the topmost – until the 20^th^ layer and measured their lipid composition.

**Figure 2:**
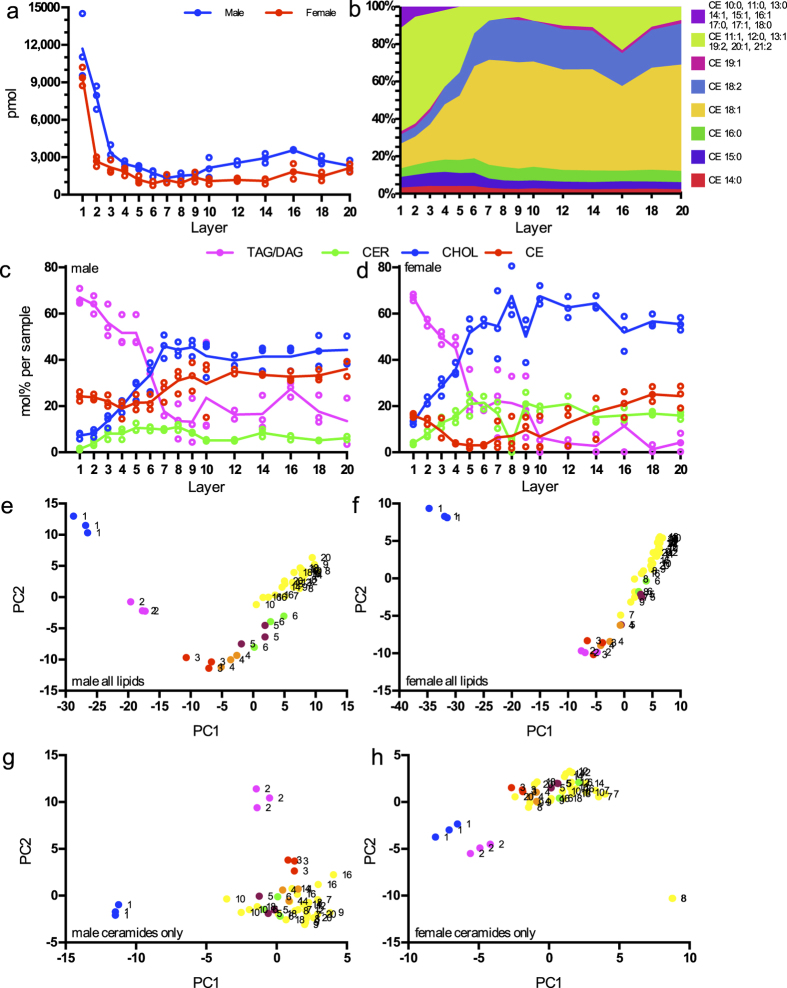
Deep sampling by sequential tape-stripping. Samples from various stratum corneum stripping layers were collected from one male and one female. (**a**) Amounts of lipids measured in different layers. Lines connect the means of each layer. (**b**) Cholesterol esters species percentage varying across layers in a male subject. (**c**) Male and (**d**) female profiles of lipid groups in different layers. CER are all ceramide classes summed up. Lines connect the means of each layer. (**e–h**) Principal component analysis of the different layer lipidomes with lipid sub-species as input. Numbers indicate layers for every replicate. (**e**) Male and (**g**) female samples with all lipids used for PCA; and (**f**) male and (**h**) female samples were only ceramides were used as input.

In both subjects we observed the total lipid amount continually decreasing by 85 and 87% until the 5^th^ and 7^th^ layer in female and male respectively, reaching a plateau thereafter (Fig. 2a). This could be explained either by a higher mass of skin sampled from the topmost layers^19^, by a higher proportion of lipids to other constituents within these layers or by a combination of both. A previous study on three subjects tape-stripped sequentially until the 20^th^ layer showed that sample weight collected by CUDERM tapes within the first layers is doubled in comparison to deeper layers^35^. However, the observed surplus of lipid amount in the uppermost layers compared to deeper layers is greater than would have resulted from the reported differences in sample weight. This suggests that the uppermost layers, while more abundant in mass, are enriched in lipids compared to deeper samples. The topical excretion of sebaceous lipids could account for this observation as sebum lipids were shown to surpass stratum corneum lipids on the skin surface over tenfold by weight^36^,^37^.

This raises the question, whether lipid classes representative of sebum penetrate into deeper stratum corneum layers. In both subjects, we observe a marked decline in the relative amount of TAGs and DAGs, jointly accounting for half of the known sebum lipids on average^38^, following the decrease in total lipid amount (Fig. 2c and d). This supports the notion of sebum lipid penetration into the SC down to the 5–7^th^ layer, where TAGs and DAGs levels reach their plateaus.

The relative amount of cholesterol and all ceramide sub-classes increased with sampling depth. Ceramides are known to be synthesized deep within SC^39^ and are not a sebum constituent, which fully accounts for the observed ceramide sampling depth gradient direction. Likewise, cholesterol constitutes as much as one third of the SC lipids, and only a small fraction of the sebum lipids^38^, which is reflected in our data in its lower relative amount at the skin surface.

Cholesterol esters were previously reported in stratum corneum^40^ as well as in sebum^38^ and this is corroborated by our measurements, where the relative amount of cholesterol esters decreased in the topmost layers and increases again below the 5–7^th^ stripping layer. Investigating the relationship between individual species of cholesterol esters and sampling depth we observed short-length fatty acid species being more abundant at or exclusive to the topmost layers (Fig. 2b). This indicates that different, preferentially shorter cholesterol ester species are of sebaceous origin, whereas others, mostly longer species stem from the stratum corneum proper.

With the principal component analyses (PCA) using all lipids as input, we were able to separate samples from the first five (female) and six layers (male) from deeper samples. Separation along principal component 1 (PC 1) corresponds to sampling depth, but samples from deeper layers do not follow this trend (Fig. 2e and f). The previously observed sebum lipid enrichment in the upper layer samples led us to postulate that mostly sebum lipids are driving the separation with respect to sampling depth. To test this hypothesis we again performed the PCA but including only ceramides as input (Fig. 2g and h). Here, the distinction of the samples by layer was limited to the first two (female) or three (male) tape-strippings.

Therefore, the above data permits the conclusion that in both subjects skin lipid composition at the volar forearm varies discernably with sampling depth. The discriminating lipids originate from the skin surface and are of sebaceous origin. The ceramide profile is less variable with respect to sampling depth.

### Intra-individual skin lipidome variability

**Figure 3:**
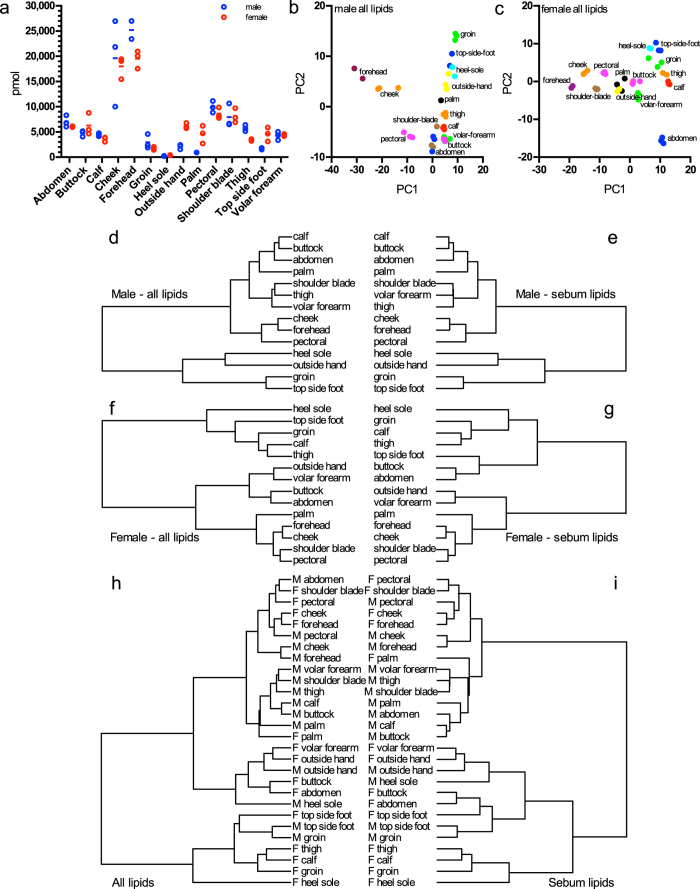
Intra-individual variability. (**a**) Amounts of lipids measured in different sampling sites. Lines connect mean values. (**b,c**) Principal component analysis of the (**b**) male and (**c**) female site lipidomes containing lipid sub-species molpercent as input. (**d–i**) Hierarchical clustering of different site lipidomes according to correlations between their lipid subspecies. (**d**) and (**e**) are male sites, with all (**d**) and only sebum lipids (**e**) used; (**f**) and (**g**) are female sites, with all (**f**) and only sebum lipids (**g**) used. (**h**) and (**i**) cluster sites from both sexes with all (**h**) and sebum only (**i**) lipids. M and F indicate sexes.

To assess intra-individual lipidome variability, samples were collected in triplicate from 14 body sites from one male and one female. Mean triplicate total lipid amount variation was 15 and 12%, for male and female respectively (Fig. 3a), which is below the variation of weight of tape-stripping as determined before (23%, Fig. 1b), indicating a general robustness of triplicate sampling across different sampling sites. Across all sampling sites we observed an intra-individual variability of total lipid amount of 104 and 81% for male and female respectively. The most distinct samples for both subjects were from forehead where the total lipid amount was as high as 21801 pmol and from heel where it was as low as 321 pmol on average. Such differences in a lipid content of skin from various body parts were observed previously^37^,^41^.

Notably, the site-specific total lipid amounts accessible to tape-stripping exhibit a significant correlation between the two subjects (Spearman correlation coefficient = 0.74, P = 0.0027). Since the likely impact of site-specific sample amounts on lipid content cannot be excluded^36^, relative lipid amounts were used to conduct PCA (Fig. 3b and c). This analysis illustrated that in both subjects the lipid profiles at any particular sampling site (i.e. within a triplicate) are more similar to one another than to lipid profiles at other sampling sites. It also indicates that skin on the forehead, cheek and to some degree from the pectoral region and shoulder blade is distinct from skin sampled from any of the other ten body sites. To confirm this distinction a hierarchical clustering was performed on averaged replicate values from all sampling sites. It shows that for both subjects cheek, forehead, pectoral region and shoulder blade lipidomes form the most closely related cluster (Fig. 3d and f). As face and back were shown to contain a high amount of triglycerides^36^ and the face features the highest density in sebum glands^42^, this led us to propose, that the facial samples, together with samples along the neckline (pectoral region and shoulder blade) are distinct from all other body sites primarily because of sebaceous lipids. Indeed, a cluster analysis of the samples considering only sebum lipids (TAGs, DAGs) confirms the same tight association of the facial samples and to some degree also the neckline samples (Fig. 3e and g). This finding holds true when investigating the similarity of both subjects jointly. Facial and neckline samples from male and female (except from the male shoulder blade) all fall into one super-cluster (Fig. 3h). Again, this similarity can be attributed fully to sebaceous lipids (Fig. 3i).

In summary, we showed the applicability of our method to skin of the entire human body. We observed site-specific differences in total lipid amounts as well as distinct lipid profiles. Skin at the investigated body sites differs predominantly by the content of sebaceous lipids, which are the major factor responsible for sample separation and clustering.

### Inter-individual variability

**Figure 4:**
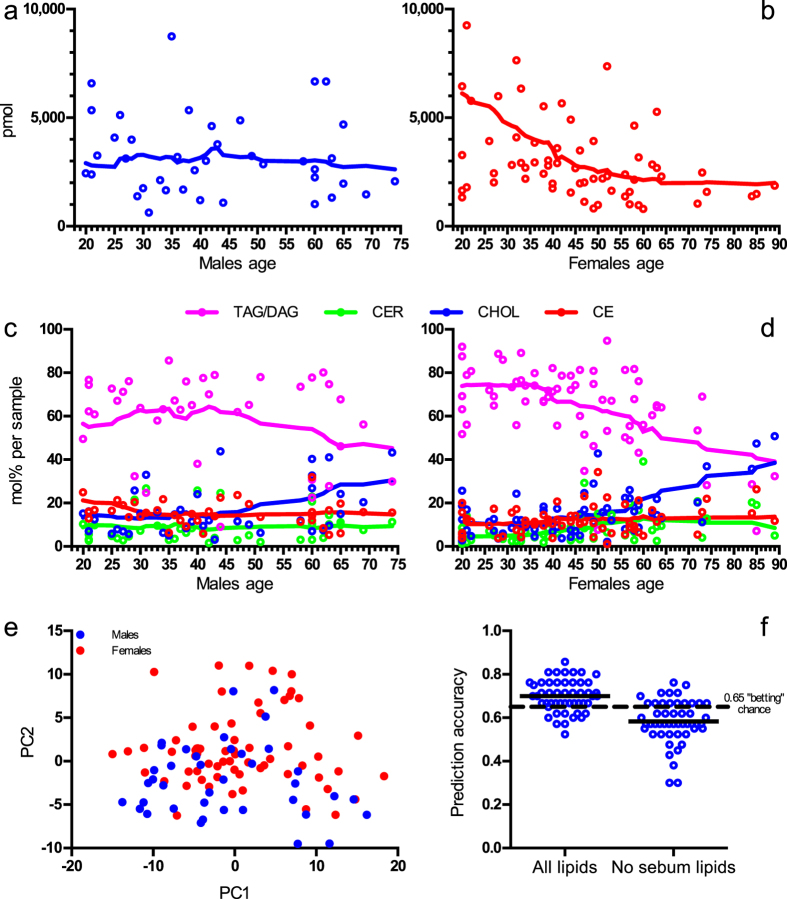
Inter-individual variation. (**a,b**) Amounts of lipids measured of (**a**) males and (**b**) females of different age (here the two most extreme pmol values are not shown to improve clarity). Lines represent 0-order polynomial smoothing function with 8 neighbours averaged. (**c,d**) Profiles of lipid groups of (**c**) males and (**d**) females of different age. Lines represent 0-order polynomial smoothing function with 8 neighbours averaged. (**e**) Principal component analysis of males and females of different age lipidomes with lipid sub-species as input. (**f**) Sex prediction accuracy (test error) of random forest classification with all lipid data and data excluding sebum lipids. Points show individual results of a 5 times repeated 10 times cross validation with horizontal lines representing their mean. The dashed line is the 0.65 Null Error Rate, the prediction error was based on always choosing the majority class.

To assess inter-individual variability 65 females and 39 males of different age were sampled at the volar forearm. In both sexes, the total lipid amount varied considerably (male: 57%, female: 90%) (Fig. 4a and b), as observed in other studies^43^. In our study, the total lipid amount in males was not correlated with age, but females, exhibited a negative correlation between age and total lipid amount (Pearson correlation coefficient = −0.40, P = 0.0009). The relative amounts of TAG and DAG in female skin markedly decreased with age, while the relative amount of cholesterol rose. This circumstance was less pronounced in the male population (Fig. 4c and d). A previous investigation of human epidermis also discovered a decline of triglycerides in older subjects, as compared with younger ones^2^, which was further corroborated by the study on 110 women where it was shown that the sebum amount decreases with age^44^. We observed relative cholesterol ester and ceramide levels remaining constant. This indifference of ceramide content to age in skin was observed before^45^. A possible explanation for gender-specific, age-dependent depletion of sebaceous lipids might be connected to hormonal regulation of sebum secretion^46^. It was shown, that at increasing age eccrine glands are either reduced in number or in functional capacity^47^. This could account for the pronounced decline of both absolute and relative amounts of sebaceous lipids among older women.

This raised the question, whether the volar forearm lipidomes of males and females are unique enough to allow sex-specific differentiation of samples. The principal component analysis showed no differences between the male and female cohorts (Fig. 4e). This finding is in line with a recent study, which did not find distinguishing features between skin ceramides of adult men and women^48^.

Also, another statistical algorithm (random forest) trained for sex differences and fitted on all samples with all lipids included, resulted in an accuracy of sex prediction based on skin lipidomes of 0.7 ± 0.073 (mean ± s.d.), while the same fitting without sebum lipids achieved only 0.58 ± 0.099 (Fig. 4f). Therefore, neither approach could generate a reliable sex prediction based on the lipidome, as a Null Error Rate is was 0.65 (where 65% of all samples are female).

In summary, our study revealed large inter-individual variability of skin lipidomes in samples collected from the volar forearm, comparable in magnitude to the intra-individual variability as measured at 14 sites on one male and one female. Both absolute and relative amounts of sebaceous lipids were observed to decrease with increasing age, especially among females.

## Discussion

In this study, we developed a method for analysis skin lipids, which combines advantages of shotgun lipidomics with benefits of tape-stripping, to produce quantitative skin lipidomes of unprecedented coverage. For the first time, stratum corneum lipids and lipids representing the majority of the sebum were analyzed simultaneously down to the level of sub-species. The linear dynamic range of the method proved sufficient to accommodate for the variability of all analyzed samples. To illustrate the capabilities of the method, we conducted a large-scale survey addressing three questions: how do skin lipid profiles change with respect to sampling depth; how variable are lipidomes of skin collected at various sampling sites of a body; and how do lipid profiles vary in volar forearm samples depending on age and gender of different subjects.

Sampling via consecutive stripping gave insight into the depth profile of sebaceous lipids down to the 20^th^ tape-stripping layer of the volar forearm skin. We observed pronounced depth dependent gradients in total lipid amounts and lipid composition. Most surprisingly, up to 5–7^th^ topmost tape-stripping layers, lipids of sebaceous origin constitute a larger part of the total lipidome than ceramides and cholesterol. In turn, in deeper samples cholesterol increases to become the most abundant lipid. Re-evaluation of the relation between lipid content and corresponding morphological features and functional parameters (transepidermal water loss, hydration), including different sampling sites, should prove insightful for establishing a role of individual lipids in skin functions.

The intra-individual variability between the 14 sites analyzed on one male and one female subject was large, both in terms of absolute lipid amounts and lipid composition. The variability within triplicate samples on the other hand was comparatively small, illustrating both method reproducibility and a local biological lipidome stability sufficient to establish lipid profiles characteristic for the surveyed sites. In both subjects, we observed that facial and neckline samples sites are more similar to one another than samples from other body regions. Similarities and differences between sampling sites were accredited to sebaceous rather than to stratum corneum lipids.

The assessment of inter-individual lipidome variability in volar forearm samples collected from 104 subjects revealed a negative correlation between total lipid amount and age for females. With increasing age the fraction of sebaceous lipids decreased, while the cholesterol fraction rose and this phenomenon was more pronounced among females then among males. However male and female samples could not be distinguished by their lipidomes.

This study raises important issues for future comparative skin lipidomic study design. Firstly, our study demonstrates the importance of the choice of sampling site and sampling depth. Depending on the objective of the study, these parameters along with age of the subjects must be controlled because their influence on measured lipidomes is dominant. Secondly, lipidome variability precludes the establishment of a general skin lipidome baseline, and comparisons should rather be made between adjacent sampling sites of the same subject. Therefore, in intervention studies topical drugs or agents should be applied to one skin site, while preferentially an adjacent, untreated site should be chosen as a negative control. These sites would then be tape-stripped and their lipidomes analyzed.

Finally, the broad lipid coverage, absolute quantification and high-throughput makes shotgun mass spectrometry based skin lipidomics a well-suited tool for rigorous and systematic studies of various topics, such as: influence of drugs on the skin lipidome; the action of substances influencing skin lipid metabolism or the skin microbiome-lipidome relation; impact of cosmetic substances on a skin lipidome with respect to their efficacy claims; and many others.

## Additional Information

**How to cite this article**: Sadowski, T. *et al*. Large-scale human skin lipidomics by quantitative, high-throughput shotgun mass spectrometry. *Sci. Rep.* **7**, 43761; doi: 10.1038/srep43761 (2017).

**Publisher's note:** Springer Nature remains neutral with regard to jurisdictional claims in published maps and institutional affiliations.

## Supplementary Material

Supplementary Information

Supplementary Dataset 1

## References

[b1] van Smeden J., Janssens M., Gooris G. S. & Bouwstra J. A. The important role of stratum corneum lipids for the cutaneous barrier function. Biochim. Biophys. Acta BBA-Mol. Cell Biol. Lipids 1841, 295–313 (2013). 10.1016/j.bbalip.2013.11.006 24252189

[b2] De Paepe K. . Analysis of Epidermal Lipids of the Healthy Human Skin: Factors Affecting the Design of a Control Population. Skin Pharmacol. Physiol. 17, 23–30 (2004). 14755124 10.1159/000074059

[b3] Ishikawa J. . Variations in the ceramide profile in different seasons and regions of the body contribute to stratum corneum functions. Arch. Dermatol. Res. 305, 151–162 (2012). 22987221 10.1007/s00403-012-1286-5

[b4] van Smeden J. . Skin barrier dysfunction in non-lesional atopic eczema: the role of stratum corneum lipids. Eur. J. Dermatol. EJD. doi: 10.1684/ejd.2013.1973 (2013). 23567090

[b5] Janssens M. . Increase in short-chain ceramides correlates with an altered lipid organization and decreased barrier function in atopic eczema patients. J. Lipid Res. 53, 2755–2766 (2012). 23024286 10.1194/jlr.P030338 PMC3494247

[b6] Paige D. G., Morse-Fisher N. & Harper J. I. Quantification of stratum corneum ceramides and lipid envelope ceramides in the hereditary ichthyoses. Br. J. Dermatol. 131, 23–27 (1994). 8043418 10.1111/j.1365-2133.1994.tb08452.x

[b7] van Smeden J. . Intercellular Skin Barrier Lipid Composition and Organization in Netherton Syndrome Patients. J. Invest. Dermatol. 134, 1238–1245 (2014). 24292773 10.1038/jid.2013.517

[b8] Elias P. M. . Localization and Composition of Lipids in Neonatal Mouse Stratum Granulosum and Stratum Corneum. J. Invest. Dermatol. 73, 339–348 (1979). 501132 10.1111/1523-1747.ep12550377

[b9] Masukawa Y. . Comprehensive quantification of ceramide species in human stratum corneum. J. Lipid Res. 50, 1708–1719 (2009). 19349641 10.1194/jlr.D800055-JLR200 PMC2724059

[b10] van Smeden J. . LC/MS analysis of stratum corneum lipids: ceramide profiling and discovery. J. Lipid Res. 52, 1211–1221 (2011). 21444759 10.1194/jlr.M014456 PMC3090242

[b11] t’Kindt R. . Profiling and Characterizing Skin Ceramides Using Reversed-Phase Liquid Chromatography–Quadrupole Time-of-Flight Mass Spectrometry. Anal. Chem. 84, 403–411 (2012). 22111752 10.1021/ac202646v

[b12] van Smeden J. . Combined LC/MS-platform for analysis of all major stratum corneum lipids, and the profiling of skin substitutes. Biochim. Biophys. Acta BBA-Mol. Cell Biol. Lipids 1841, 70–79 (2014). 10.1016/j.bbalip.2013.10.002 24120918

[b13] Wu Z. . Lipidomic platform for structural identification of skin ceramides with α-hydroxyacyl chains. Anal. Bioanal. Chem. 408, 2069–2082 (2016). 26815554 10.1007/s00216-015-9239-4

[b14] Shin J.-H. . A lipidomic platform establishment for structural identification of skin ceramides with non-hydroxyacyl chains. Anal. Bioanal. Chem. 406, 1917–1932 (2014). 24458481 10.1007/s00216-013-7601-y

[b15] Sandra K. & Sandra P. Lipidomics from an analytical perspective. Curr. Opin. Chem. Biol. 17, 847–853 (2013). 23830914 10.1016/j.cbpa.2013.06.010

[b16] Surma M. A. . An automated shotgun lipidomics platform for high throughput, comprehensive, and quantitative analysis of blood plasma intact lipids. Eur. J. Lipid Sci. Technol. 117, 1540–1549 (2015). 26494980 10.1002/ejlt.201500145 PMC4606567

[b17] Bonté F., Pinguet P., Chevalier J. M. & Meybeck A. Analysis of all stratum corneum lipids by automated multiple development high-performance thin-layer chromatography. J. Chromatogr. B. Biomed. Sci. App. 664, 311–316 (1995). 10.1016/0378-4347(94)00480-s 7780582

[b18] Motta S. . Ceramide composition of the psoriatic scale. Biochim. Biophys. Acta 1182, 147–151 (1993). 8357845 10.1016/0925-4439(93)90135-n

[b19] Breternitz M., Flach M., Prässler J., Elsner P. & Fluhr J. W. Acute barrier disruption by adhesive tapes is influenced by pressure, time and anatomical location: integrity and cohesion assessed by sequential tape stripping. A randomized, controlled study. Br. J. Dermatol. 156, 231–40 (2007). 17223861 10.1111/j.1365-2133.2006.07632.x

[b20] Liebisch G., Drobnik W. & Reil M. Quantitative measurement of different ceramide species from crude cellular extracts by electrospray ionization tandem mass spectrometry (ESI-MS/MS). J. Lipid Res. 40, 1539–1546 (1999). 10428992

[b21] Schwudke D. . Top-down lipidomic screens by multivariate analysis of high-resolution survey mass spectra. Anal. Chem. 79, 4083–4093 (2007). 17474710 10.1021/ac062455y

[b22] Han X. & Gross R. W. Shotgun lipidomics: Electrospray ionization mass spectrometric analysis and quantitation of cellular lipidomes directly from crude extracts of biological samples. Mass Spectrom. Rev. 24, 367–412 (2005). 15389848 10.1002/mas.20023

[b23] Schuhmann K. . Bottom-up shotgun lipidomics by higher energy collisional dissociation on LTQ Orbitrap mass spectrometers. Anal. Chem. 83, 5480–5487 (2011). 21634439 10.1021/ac102505f

[b24] Herzog R. . LipidXplorer: a software for consensual cross-platform lipidomics. PloS One 7, e29851 (2012). 22272252 10.1371/journal.pone.0029851 PMC3260173

[b25] Weissgerber T. L., Milic N. M., Winham S. J. & Garovic V. D. Beyond Bar and Line Graphs: Time for a New Data Presentation Paradigm. PLOS Biol. 13, e1002128 (2015). 25901488 10.1371/journal.pbio.1002128 PMC4406565

[b26] Kuhn M. Building Predictive Models in *R* Using the **caret** Package. J. Stat. Softw. 28 (2008).

[b27] Liaw A. & Wiener M. Classification and regression by randomForest. R News 2, 18–22 (2002).

[b28] Jung H., Sylvänne T. & Koistinen K. High throughput quantitative molecular lipidomics. Biochim. Biophys. Acta BBA-Mol. Cell Biol. Lipids 1811, 925–934 (2011). 10.1016/j.bbalip.2011.06.025 21767661

[b29] van der Molen R. G. . Tape stripping of human stratum corneum yields cell layers that originate from various depths because of furrows in the skin. Arch. Dermatol. Res. 289, 514–518 (1997). 9341971 10.1007/s004030050232

[b30] Folch J., Lees M. & Sloane-Stanley G. H. & others. A simple method for the isolation and purification of total lipids from animal tissues. J Biol Chem 226, 497–509 (1957). 13428781

[b31] Bligh E. G. & Dyer W. J. A rapid method of total lipid extraction and purification. Can. J. Biochem. Physiol. 37, 911–917 (1959). 13671378 10.1139/o59-099

[b32] Matyash V., Liebisch G., Kurzchalia T. V., Shevchenko A. & Schwudke D. Lipid extraction by methyl-tert-butyl ether for high-throughput lipidomics. J. Lipid Res. 49, 1137–1146 (2008). 18281723 10.1194/jlr.D700041-JLR200 PMC2311442

[b33] Heiskanen L. A., Suoniemi M., Ta H. X., Tarasov K. & Ekroos K. Long-term performance and stability of molecular shotgun lipidomic analysis of human plasma samples. Anal. Chem. 85, 8757–8763 (2013). 23919256 10.1021/ac401857a

[b34] Booth B. . Workshop Report: Crystal City V—Quantitative Bioanalytical Method Validation and Implementation: The 2013 Revised FDA Guidance. AAPS J. 17, 277–288 (2015). 25549614 10.1208/s12248-014-9696-2 PMC4365089

[b35] Dreher F. . Colorimetric method for quantifying human stratum corneum removed by adhesive-tape-stripping. Acta Derm. Venereol. 78, 186–189 (1998). 9602223 10.1080/000155598441495

[b36] Greene R. S., Downing D. T., Pochi P. E. & Strauss J. S. Anatomical Variation in the Amount and Composition of Human Skin Surface Lipid. J. Invest. Dermatol. 54, 240–247 (1970). 5436951 10.1111/1523-1747.ep12280318

[b37] Lampe M. A. . Human stratum corneum lipids: characterization and regional variations. J. Lipid Res. 24, 120–130 (1983). 6833889

[b38] Pappas A. Epidermal surface lipids. Dermatoendocrinol. 1, 72–76 (2009). 20224687 10.4161/derm.1.2.7811 PMC2835894

[b39] Rabionet M., Gorgas K. & Sandhoff R. Ceramide synthesis in the epidermis. Biochim. Biophys. Acta 1841, 422–434 (2013). 23988654 10.1016/j.bbalip.2013.08.011

[b40] Lavrijsen A. P. M. . Validation of an *in vivo* extraction method for human stratum corneum ceramides. Arch. Dermatol. Res. 286, 495–503 (1994). 7864665 10.1007/BF00371579

[b41] Pagnoni A., Kligman A. M., Gammal S. e. & Stoudemayer T. Determination of density of follicles on various regions of the face by cyanoacrylate biopsy: correlation with sebum output. Br. J. Dermatol. 131, 862–865 (1994). 7857840 10.1111/j.1365-2133.1994.tb08590.x

[b42] Mills O. H. Jr. & Kligman A. M. The Follicular Biopsy. Dermatology 167, 57–63 (1983). 10.1159/000249749 6226549

[b43] Norlén L., Nicander I., Rozell B. L., Ollmar S. & Forslind B. Inter- and Intra-Individual Differences in Human Stratum Corneum Lipid Content Related to Physical Parameters of Skin Barrier Function *In Vivo*. J. Invest. Dermatol. 112, 72–77 (1999). 9886267 10.1046/j.1523-1747.1999.00481.x

[b44] Wendling P.-A. & Dell’Acqua G. Skin biophysical properties of a population living in Valais, Switzerland. Skin Res. Technol. 9, 331–338 (2003). 14641883 10.1034/j.1600-0846.2003.00040.x

[b45] Denda M. . Age- and sex-dependent change in stratum corneum sphingolipids. Arch. Dermatol. Res. 285, 415–417 (1993). 8304781 10.1007/BF00372135

[b46] Thiboutot D. Regulation of Human Sebaceous Glands. J. Invest. Dermatol. 123, 1–12 (2004). 15191536 10.1111/j.1523-1747.2004.t01-2-.x

[b47] Fenske N. A. & Lober C. W. Structural and functional changes of normal aging skin. J. Am. Acad. Dermatol. 15, 571–585 (1986). 3534008 10.1016/s0190-9622(86)70208-9

[b48] Jia Z.-X., Zhang J.-L., Shen C.-P. & Ma L. Profile and quantification of human stratum corneum ceramides by normal-phase liquid chromatography coupled with dynamic multiple reaction monitoring of mass spectrometry: development of targeted lipidomic method and application to human stratum corneum of different age groups. Anal. Bioanal. Chem. 408, 6623–6636 (2016). 27473427 10.1007/s00216-016-9775-6

