# Supplementary material for: Large-scale human skin lipidomics by quantitative, high-throughput shotgun mass spectrometry
Source: Sci Rep. 2017 Mar 7;7:43761. doi: 10.1038/srep43761 (PMC5339821; doi:10.1038/srep43761)
Supplement: Supplementary Information [file srep43761-s1.pdf]

Large-scale human skin lipidomics by quantitative, high-throughput shotgun mass spectrometry

Tomasz Sadowski[1,2], Christian Klose[2], Mathias J. Gerl[2], Anna Wójcik-Maciejewicz[3], Ronny Herzog[2], Kai Simons[2,4], Adam Reich[3], Michal A. Surma[2]*

[1]Medical Faculty, Dresden University of Technology, Dresden, Germany

[2]Lipotype GmbH, Dresden, Germany

[3]Department of Dermatology, Venerology and Allergology, University of Medicine, Wroclaw, Poland.

[4]Max Planck Institute for Molecular Cell Biology and Genetics, Dresden, Germany

*Correspondence and requests for materials should be addressed to MAS (email: surma@lipotype.com)

Lipid class	Acquisition mode	Precursor ion	Fragment ion	Structural detail	Example	slope/r^2
Cholesterol (Chol)	Pos FTMS	$[MC_2H_2O_2+NH_4^+]^+$	-	Class	Chol	1.05/1.00
Triacylglycerol (TAG)	Pos FTMS/MSMS	$[M+NH_4^+]^+$	$[M\text{-}FA]^+$	Species	TAG 48:2;0	0.85/1.00
Diacylglycerol (DAG)	Pos FTMS/MSMS	$[M+NH_4^+]^+$	$[M\text{-}FA]^+$	Sub-species	DAG 38:2;0 (18:2;0-20:0:0)	0.91/1.00
Cholesterol esters (CE)	Pos FTMS/MSMS	$[M+NH_4^+]^+$	$[FA\text{-}H^+]^-$	Species	CE 18:2;0	0.89/0.99
Non-hydroxy-dehydrosphingosine (NdS)	Neg FTMS/MSMS	$[M+C_2H_3O_2+H^-]^-$	$[FA\text{-}H^+]^-$	Sub-species	NdS 44:0;2 (20:0;2-24:0;0)	nd
Non-hydroxy-sphingosine (NS)	Neg FTMS/MSMS	$[M+C_2H_3O_2+H^-]^-$	$[FA\text{-}H^+]^-$	Sub-species	NS 44:1;2 (18:1;2-26:0;0)	0.96/0.99
Non-hydroxy-phytosphingosine (NP)	Neg FTMS/MSMS	$[M+C_2H_3O_2+H^-]^-$	$[FA\text{-}H^+]^-$	Sub-species	NP 46:0;3 (20:0;3-26:0;0)	nd
Non-hydroxy-6-hydroxy-sphingosine (NH)	Neg FTMS/MSMS	$[M+C_2H_3O_2+H^-]^-$	$[FA\text{-}H^+]^-$	Sub-species	NH 46:1;3 (18:1;3-28:0;0)	nd
Alphahydroxy-dehydrosphingosine (AdS)	Neg FTMS/MSMS	$[M+C_2H_3O_2+H^-]^-$	$[FA\text{-}H^+]^-$	Sub-species	AdS 44:0;3 (19:0;2-25:0;1)	nd
Alphahydroxy-sphingosine (AS)	Neg FTMS/MSMS	$[M+C_2H_3O_2+H^-]^-$	$[FA\text{-}H^+]^-$	Sub-species	AS 44:1;3 (18:1;2-26:0;1)	nd
Alphahydroxy-phytosphingosine (AP)	Neg FTMS/MSMS	$[M+C_2H_3O_2+H^-]^-$	$[FA\text{-}H^+]^-$	Sub-species	AP 42:0;4 (18:0;3-24:0;1)	nd
Alphahydroxy-6-hydroxysphingosine (AH)	Neg FTMS/MSMS	$[M+C_2H_3O_2+H^-]^-$	$[FA\text{-}H^+]^-$	Sub-species	AH 44:1;4 (18:1;3-26:0;1)	nd
Omegahydroxy-dehydrosphingosine (EOdS)	Neg FTMS/MSMS	$[M+C_2H_3O_2+H^-]^-$	[linoleic acid-H^+]$^-$	Species	EOdS 68:2;2	nd
Omegahydroxy-sphingosine (EOS)	Neg FTMS/MSMS	$[M+C_2H_3O_2+H^-]^-$	[linoleic acid-H^+]$^-$	Species	EOS 68:3;2	1.21/0.99
Omegahydroxy-phytosphingosine (EOP)	Neg FTMS/MSMS	$[M+C_2H_3O_2+H^-]^-$	[linoleic acid-H^+]$^-$	Species	EOP 68:2;3	nd
Omegahydroxy-6-hydroxy-sphingosine (EOH)	Neg FTMS/MSMS	$[M+C_2H_3O_2+H^-]^-$	[linoleic acid-H^+]$^-$	Species	EOH 68:3;3	nd

Supplementary Table 1. Lipid classes covered by the method, with modes of their acquisition, ions used for their identification, confirmation and in MSMS-fragmentation scan. M – molecular ion, FA – fatty acid.

Dataset 1. Complete dataset of the lipid composition of skin samples analyzed (pmol/sample). Available as separate file (*.xls).
